# Effect of Low pH and Aluminum Toxicity on the Photosynthetic Characteristics of Different Fast-Growing *Eucalyptus* Vegetatively Propagated Clones

**DOI:** 10.1371/journal.pone.0130963

**Published:** 2015-06-19

**Authors:** Mei Yang, Ling Tan, Yuanyuan Xu, Yihui Zhao, Fei Cheng, Shaoming Ye, Weixin Jiang

**Affiliations:** 1 Forestry College, Guangxi University, Nanning, Guangxi, China; 2 Key Lab of National Forestry Bureau for Fast-growing Wood Breeding in Central South China, Guangxi University, Nanning, Guangxi, China; 3 Guangxi Colleges and Universities Key Laboratory of Forestry Science and Engineering, Nanning, Guangxi, China; Universidade Federal de Vicosa, BRAZIL

## Abstract

Knowing how acid soils and aluminum in soils may limit the growth of *Eucalyptus* trees in plantations is important because these plantations grow in many tropical and subtropical regions. Seedlings of four vegetatively propagated *Eucalyptus* clones, *E*. *grandis × E*. *urophylla* ‘GLGU9’(G9), *E*. *grandis × E*. *urophylla* ‘GLGU12’ (G12), E. *urophylla × E*. *camaldulensis* ‘GLUC3’ (G3) and *E*. *urophylla* ‘GLU4’(G4), were subjected to liquid culture with Hoagland nutrient solution for 40 days, then treated with four different treatments of acid and aluminum for 1 day. The four treatments used either pH 3.0 or 4.0 with or without added aluminum (4.4 mM) in all possible combinations; a control used no added aluminum at pH 4.8. Subsequently, the photosynthetic parameters and morphology of leaves from eucalypt seedlings were determined and observed. The results showed that the tested chlorophyll content, net photosynthetic rate, transpiration rate and water use efficiency were apparently inhibited by aluminum. Under uniform Al concentration (4.4 mM), the Al-induced limitation to photosynthetic parameters increased with pH, indicating acid stimulation to Al toxicity. Among all treatments, the most significant reduction was found in the combination of pH 3.0 and 4.4 mM Al. The photosynthetic and transpiration rates showed similar trends with G9 > G12 > G3 > G4, suggesting that G9 and G12 had higher Al-tolerance than other two clones. Microscopic observation revealed changes in leaf morphology when exposed to Al stress; for example, a reduced thickness of leaf epidermis and palisade tissue, the descendant palisade tissue/spongy tissue ratio and leaf tissue looseness. Overall, the acid and aluminum stress exerted negative effects on the photosynthetic activity of eucalypt seedlings, but the differences in tolerance to Al toxicity between the clones were favorable, offering potential to improve *Eucalyptus* plantation productivity by selecting Al tolerant clones.

## Introduction

Aluminum is the most abundant metal element in the earth’s crust and bound aluminum will dissolve in acidic soils. When soluble A1^3+^ content reaches 10~20 mg/kg or more, it produces severe toxic effects on plants [1, 2]. For example, aluminum can cause oxidative stress by increase in production of reactive oxygen species (ROS) which may affect unsaturated fatty acids in cell membrane, indirectly affecting plant metabolism [3–5]. In addition, aluminum also can replace calcium to interfere with cellular expansion processes [6]. Currently, aluminum resistance mechanisms are classified into exclusion and tolerance [3], both mechanisms are related to mitochondrial activity [7] as well as organic acid transport [8, 9]. For exclusion mechanism, aluminum detoxication happens in plant rhizospheres through the exudation of organic acid anions. The organic acid anions (malate, citrate and oxalate) are released by plants to chelate the toxic Al^3+^ in the rhizosphere, such resistance mechanism to Al toxicity has been observed in crops, such as wheat, barley, rye, rice and maize. For woody plants, both coniferous (*Picea*, *Cryptomeria* and *Pinus*) and broad-leaved (*Populus*, *Eucalyptus*, *Citrus* and *Melaleuca*) trees also have this mechanism [8]. Several genes have been demonstrated to be responsible for encoding transporters for organic acid anions, or be up- or down- regulated by aluminum stress [3, 9, 10]. More organic acid anions are usually exuded by tolerant genotypes than sensitive ones. Researches related to the aluminum tolerance of different genotypes of a single crop species revealed that high concentrations of aluminum limited root growth, causing the accumulation of reactive oxygen species [11, 12], and causing damage to DNA [12] in aluminum-sensitive genotypes.

Al not only affects plant roots, but photosynthetic behavior also is subjected to Al toxicity. For Al-sensitive plants, present of Al may reduce stomatal conductance [13] and chlorophyll content[13–15], change chlorophyll a/b ratio [16], photosynthetic rate usually declines[14, 16, 17], electron transport is inhibited [18, 19], but transpiration and water use efficiency are not always to reduce [13, 17, 20, 21]. These variations are not significant in Al-tolerant plants [17, 19, 22, 23], Al toxicity symptoms in photosynthetic activity has a close linkage with Al concentration in environments [13, 24]. Currently, most of knowledge on photosynthetic responses to Al is primarily from fruit trees, tomatoes, maize, soybeans and other cereal crops, there is a gap in woody plants. Studying the ability of woody plants to tolerate high levels of soil aluminum, and breeding and cultivating crop varieties with aluminum tolerance is important to enhance the productive potential of acidic soil and improve crop yield, for both agriculture and forestry.

Acidic soil covers more than 40 percent of the world’s arable land; China ranks third in countries experiencing acid rain followed by Europe and North America. Acid deposition exacerbates South China’s problems related to acidic, aluminum-rich soil conditions. The effects of aluminum toxicity on plants have drawn wide attention. However, little relevant research has been was conducted regarding the effect of low pH; the study of the effect of low pH on plants has an important role for further evaluation of the combined effects of pH and aluminum toxicity on plants [25].

*Eucalyptus* is extremely important fast-growing plantation species in tropical and subtropical regions, however, decreased productivity has been observed in short-rotation plantation forests. This may be caused by acid/aluminum toxicity. For example, because of the potential risk of aluminum toxicity in soils of eucalypt forests in the Santiago de Compostela and Lugo regions of Spain, a study was undertaken. It showed that the aluminum content in soil and accumulation of aluminum in eucalypt roots were obviously affected by soil acidity [26]. Other studies used hydroponics to demonstrate that aluminum stress suppressed the growth and physiological processes of eucalypt and that the adaptability of eucalypt to Al was related to the genotypes [27–30]. Currently, eucalypts are propagated vegetatively; therefore, studying the responses of different clones (genotypes) to acid/aluminum stress and screening clones for aluminum-tolerate fast-growing eucalypt individuals are of great significance. Different *Eucalyptus* species may release distinctive organic acid anions following Al^3+^ exposure, *E*. *urophylla* and *E*. *dunnii* for citrate, malate and oxalate, *E*. *camaldulensis* for citrate and oxalate, *E*. *globulus* for citrate and malate, *E*. *cloeziana*, *E*. *grandis* and *E*. *saligna* for citrate [8, 28, 29, 31]. Yang et al. [32, 33] performed an assessment of aluminum tolerance on growth performance and stress physiological parameters of four fast-growing eucalypt clones and found their Al^3+^ tolerance can be classified as *E*. *grandis × E*. *urophylla* ‘GLGU9’, *E*. *grandis × E*. *urophylla* ‘GLGU12’, *E*. *urophylla × E*. *camaldulensis* ‘GLUC3’ and *E*. *urophylla* ‘GLU4’. This study compared the changes of photosynthetic characteristics of four fast-growing eucalypt clones under acid/aluminum treatments to provide a reference for evaluating aluminum tolerance of different clones and to provide a theoretical basis to further reveal the relationship between acid/aluminum toxicity and photosynthesis of trees.

## Materials and Methods

### Study sites

This study was conducted at the Guangxi University in Nanning, Guangxi Province, China. Nanning is located in the south of the tropic, the average altitude is 74~79 m, the geographic coordinates are N22°13′~23°32′ in latitude and E107°45′~108°51′ in longitude. Nanning belongs to the subtropical monsoon climate, the annual average temperature is 21.6°C, the annual precipitation 1304.2 mm, the average relative humidity 79%, the frost-free period 345~360 days. The topography of Nanning is composed of flatlands, low mountains, stone mountains, hills and bench terraces, the prevailing soil type is latosolic red soil.

### Ethics Statement

No specific permissions were required for the locations/activities, the location was not privately-owned or protected in any way, the used plant materials were not endangered or protected species.

### Experimental design

This study was conducted at the Guangxi University of China. Four clones: *E*. *grandis × E*. *urophylla* ‘GLGU9’, *E*. *grandis × E*. *urophylla* ‘GLGU12’, *E*. *urophylla × E*. *camaldulensis* ‘GLUC3’ and *E*. *urophylla* ‘GLU4’ were used as the experimental materials (labeled as G9, G12, G3 and G4, respectively). The seedlings used were two month-old tissue cultured plantlets. Healthy and uniformly-sized seedlings were cultured in sterilized plastic buckets (12 seedlings/bucket) containing 2 L filter-sterilized Hoagland nutrient solution. The Hoagland nutrient solution contained 101.1 mg/L KNO_3_, 164.2 mg/L Ca(NO_3_)_2_, 48.2 mg/L MgSO_4_, 23.0 mg/L NH_4_H_2_PO_4_, 3.7 mg/L Fe-EDTA, 1.24 mg/L H_3_BO_3_, 0.60 mg/L MnCl_2_, 0.32 mg/L ZnSO_4_, 0.08 mg/L CuSO_4_ and 0.47 mg/L (NH_4_)_6_Mo_7_O_24_. The filter-sterilized nutrient solution was replaced once every two days. Prior to replacement, the seedlings were put into a plastic bucket with 0.1% carbendazim for 20 minutes for inhibiting pathogenic microorganisms. During culture, air pumps were used to aerate (50 L/h).

After 40 days of liquid culture, the seedlings received one of four treatments using AlCl_3_ × 6H_2_O as follows: K_0-3_ treatment, 0 mM Al^3+^, pH 3.0; K_4.4–3_ treatment, 4.4 mM Al^3+^, pH 3.0; K_0-4_ treatment, 0 mM Al^3+^, pH 4.0; K_4.4–4_ treatment, 4.4 mM Al^3+^, pH 4.0; control treatment (CK), 0 mM Al^3+^, pH 4.8. In a previous field investigation, soil soluble Al concentration of 5-year-old plantations was 4.4 mM on average, therefore this concentration was selected in this study. Each treatment had three replicates. The seedlings were treated with acid and Al in a new and filter-sterilized Hoagland nutrient solution which contained 0.5 mM CaCl_2_. At the same time of replacing the nutrient solution, AlCl_3_ was added, pH was adjusted with 2 mol/L HCl or NaOH until reaching pH 3.0 or 4.0. One day after treatment, the second and third fully expanded leaves from the top of the seedlings were harvested for measurement of chlorophyll content, photosynthetic and transpiration rates, and for observation of leaf morphology. These leaves were selected as young leaves are more sensitive to environmental stress than older ones, hence any Al-induced effects could be detected [32].

### Determination of the photosynthetic parameters of leaves

The photosynthesis parameters of leaves were measured using a CI-340 photosynthesis analyzer (CID Inc., Camus, WA, USA), the determination steps referred to manufacturer’s protocol. The net photosynthetic rates were measured using low to high light intensity, allowing the confirmation of the light compensation point (15.288 μmol × m^−2^ × s^-1^) and light saturation point (1193.580 μmol × m^-2^ × s^-1^). Therefore, the following settings were used: light intensity of the leaf room for the photosynthetic measurement system, 1200 μmol m^-2^ s^-1^; temperature, 25±1°C; relative humidity, 70%. Then, the net photosynthetic and transpiration rates as well as water use efficiency (WUE = Pn/Tr) (μmol CO_2_/mmol H_2_O) were measured. Chlorophyll was extracted by the acetone and ethanol immersion method [34]; chlorophyll absorbance was measured using a microplate reader (UNAGUBG-PAM, WALZ, Germany).

### Observations of leaf morphology

For the study of leaf morphology, fresh green leaves near the top of seedlings were harvested. Several 3 × 5 mm pieces in the middle of leaf stem were cut and held with fresh moist carrot and a thin slice was made for examination with a microscope. Photographs were made of the thickness of the upper and lower epidermis, and the thickness of the palisade and spongy tissues of the leaf. These were observed and measured at 100 × magnification under an optical microscope (DMBA300-A, China). Five slices were made for leaves from each treatment, with ten photographs taken for each slice for measurement. Calculations were made for the leaf palisade and spongy tissue ratio (P/I), organizational tightness (CTR) and tissue porosity (SR).

### Data analysis

The experimental design was assigned as 4 clones × 5 treatments (including CK) × 3 replicates (buckets). All data between different treatments was compared using one-way ANOVA with LSD’s post-hoc test. The results were presented in the form of mean±standard deviation. In the following figures and table, capital letters represent significant differences between treatments within an eucalypt clone at the 0.01 level, small letters are at the 0.05 level, n = 3. All statistical and graphical processes were performed with R and relevant packages. All collected raw data of this study have been provided in S1 File.

## Results

### Photosynthetic parameters

#### Chlorophyll content

**Fig 1:**
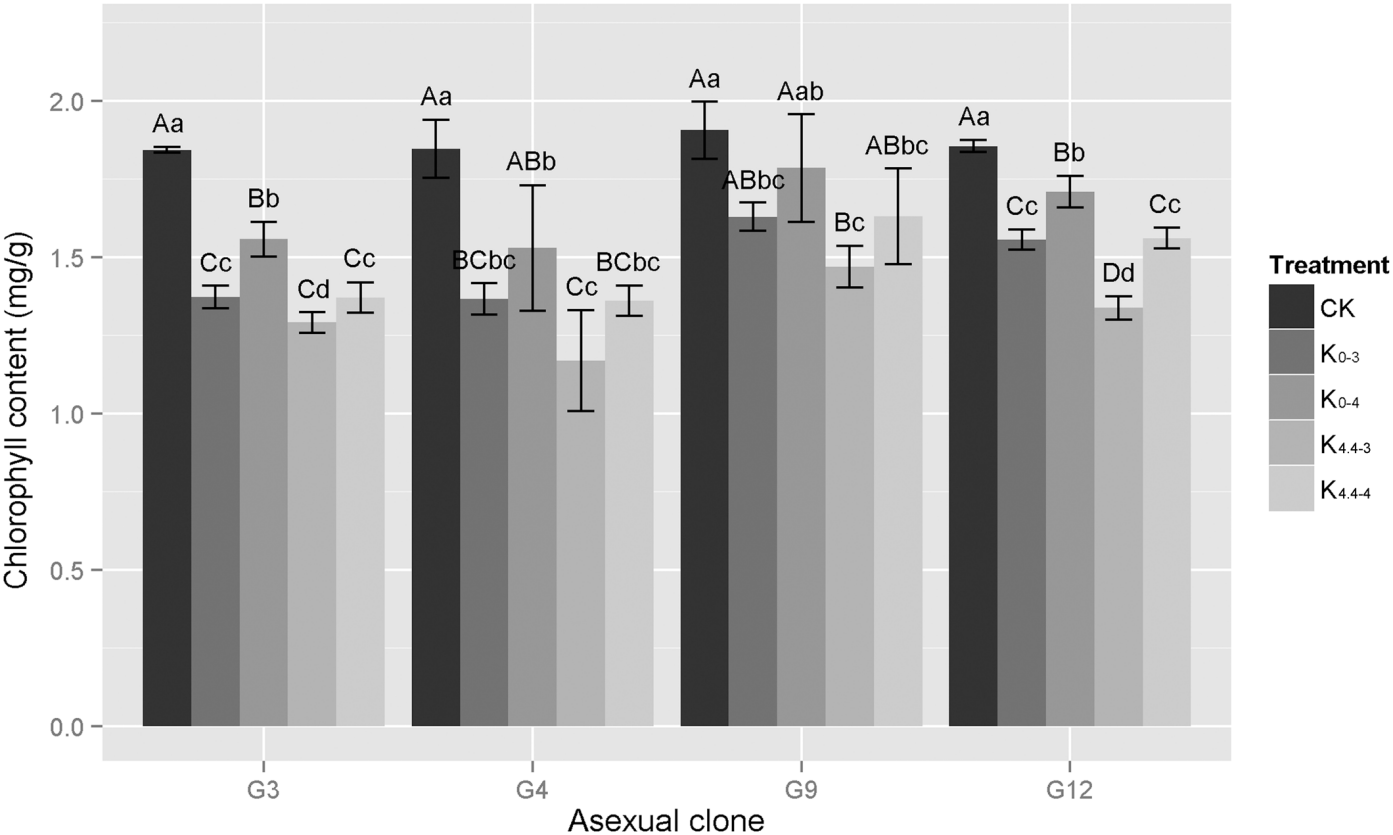
Changes in seedling chlorophyll content of four eucalypt clones under different acid aluminum treatments. G3 represents *E*. *urophylla × E*. *camaldulensis* ‘GLUC3’; G4, *E*. *urophylla* ‘GLU4’; G9, *E*. *grandis × E*. *urophylla* ‘GLGU9’; G12, *E*. *grandis × E*. *urophylla* ‘GLGU12’. Treatments: K_0-3_, treatment with 0 mM Al^3+^, pH 3.0; K_4.4–3_, 4.4 mM Al^3+^, pH 3.0; K_0-4_, 0 mM Al^3+^, pH 4.0; K_4.4–4_, 4.4 mM Al^3+^, pH 4.0; CK, control treatment, 0 mM Al^3+^, pH 4.8. Capital letters represent significant differences between treatments within an eucalypt clone at the 0.01 level, small letters are at the 0.05 level, n = 3.

Different acid aluminum treatments, including strong acids and high concentrations of aluminum, cause toxicity in *Eucalyptus* leaves, in many cases destroying the chloroplast envelopes and resulting in decreased chlorophyll content (Fig 1). Under constant acidity, the chlorophyll content of identical clones without aluminum treatment was higher than that with aluminum treatment (K_0-3_ > K_4.4–3_, K_0-4_ > K_4.4–4_). Under constant aluminum concentrations, increased acidity resulted in lower chlorophyll content (K_0-3_ < K_0-4_, K_4.4–3_ < K_4.4–4_). The difference in chlorophyll content between the K_0-3_ and K_4.4–4_ treatments within the same clone was not significant. In general, the chlorophyll content in the clones in descending order was: G9 > G12 > G3 > G4. Compared with the CK, the chlorophyll content in various clones decreased after acid aluminum treatment with the K_0-4_ treatment causing the largest decline and the K_4.4–3_ treatment causing the smallest. The declines in chlorophyll content for G3, G4, G9 and G12 were 29.9%, 36.7%, 23.0%, 27.9% and 15.5%, 17.2%, 6.5%, 7.9% in the K_0-4_ and K_4.4–3_ treatments, respectively. Thus, the decline in G9 and G12 were two times lower than those in G3 and G4, and there was a narrower gap between the four clones with the degree of stress increased.

#### Net photosynthetic rate

**Fig 2:**
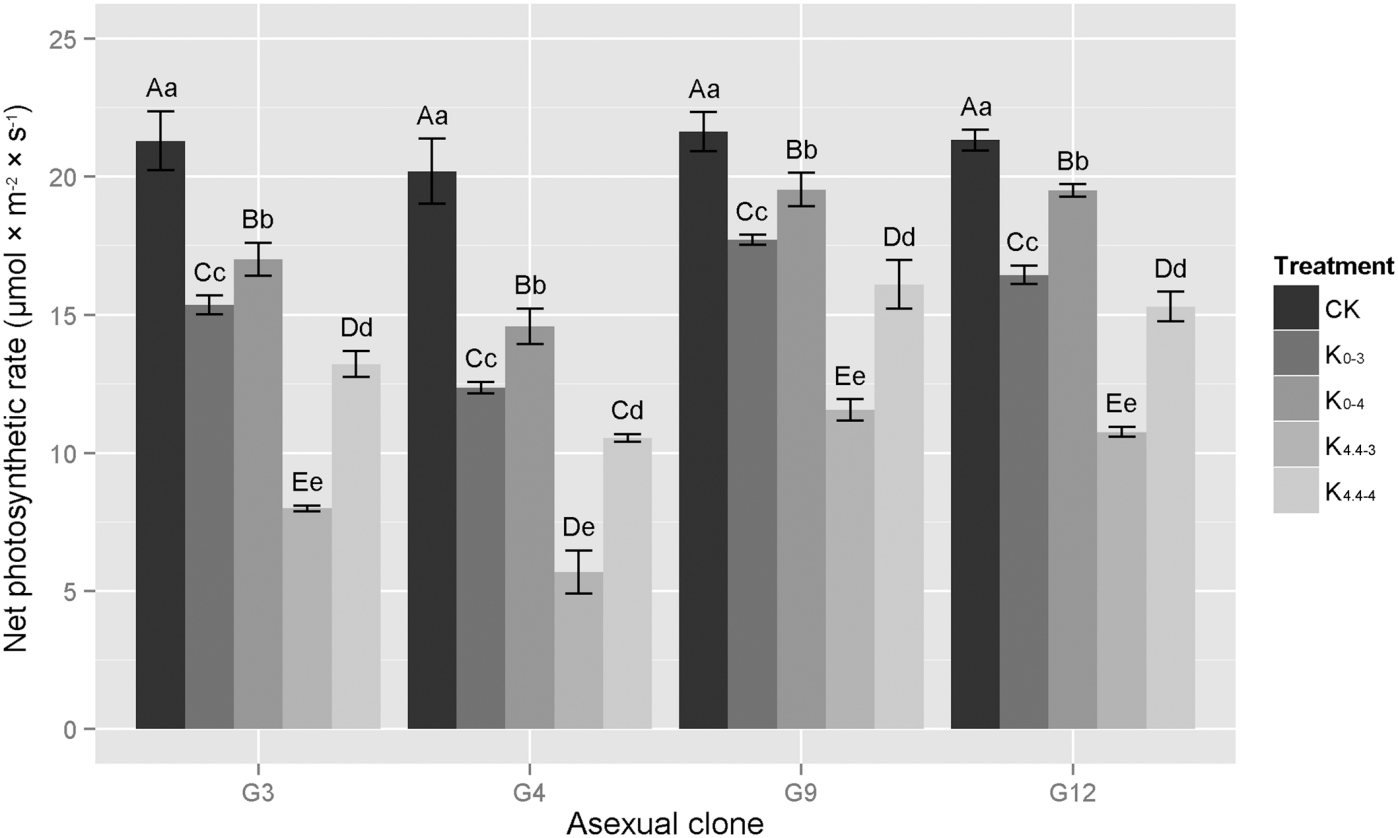
Changes in net photosynthetic rates of four eucalypt clones under different acid aluminum treatments. G3 represents *E*. *urophylla × E*. *camaldulensis* ‘GLUC3’; G4, *E*. *urophylla* ‘GLU4’; G9, *E*. *grandis × E*. *urophylla* ‘GLGU9’; G12, *E*. *grandis × E*. *urophylla* ‘GLGU12’. Treatments: K_0-3_, treatment with 0 mM Al^3+^, pH 3.0; K_4.4–3_, 4.4 mM Al^3+^, pH 3.0; K_0-4_, 0 mM Al^3+^, pH 4.0; K_4.4–4_, 4.4 mM Al^3+^, pH 4.0; CK, control treatment, 0 mM Al^3+^, pH 4.8. Capital letters represent significant differences between treatments within an eucalypt clone at the 0.01 level, small letters are at the 0.05 level, n = 3.

Under acid aluminum treatments, the photosynthetic rates in four eucalypt clones gradually decreased with the increase of the intensity of stress, although significant differences were observed between the treatments in each clone. The photosynthetic rates ofclones at pH 4.0 were greater than those at pH 3.0, and after the addition of Al^3+^, photosynthesis was inhibited and occurred at lower rates than in those treatments without aluminum (Fig 2). The photosynthetic rate was lower in the K_4.4–4_ treatment than the K_0-3_ treatment, indicating aluminum ions further weakened and interfered with the process of photosynthesis. The photosynthetic rate was lowest for each clone of the K_4.4–3_ treatment. The photosynthetic rate in G3 and G4 were 8 and 5.69 μmol × m^-2^ × s^-1^, respectively, lower than 10 μmol × m^-2^ × s^-1^, while the photosynthetic rate in G9 and G12 were slightly higher than 10 μmol × m^-2^ × s^-1^, at 11.55 and 10.76 μmol × m^-2^ × s^-1^, respectively.

Through the analysis of changes in the magnitude of the net photosynthetic rates in the four clones, we found that both the CK or the acid/aluminum treatment, the decline in G9 and G12 was always lower than that in G3 and G4. Meanwhile, under the acid/aluminum treatments, the decline in net photosynthetic rates in G3 and G4 was about 20% higher than those in G9 and G12. Especially in the case of K_0-4_, the declines in G9 and G12 were only 9.7% and 8.6%, respectively, while the declines in G3 and G4 were 20.2% and 27.9%, respectively. Meanwhile, in the case of K_4.4–3_, the declines were largest in G3, G4, G9, and G12, or 62.4%, 71.8%, 46.6% and 49.6%, respectively. Photosynthesis was severely affected in the G4 treatment.

#### Transpiration rate

**Fig 3:**
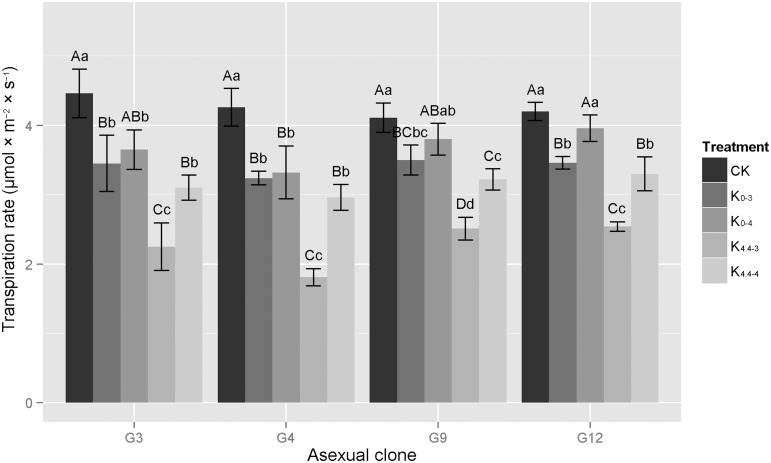
Changes in transpiration rates of four eucalypt clones under different acid aluminum treatments. G3 represents *E*. *urophylla × E*. *camaldulensis* ‘GLUC3’; G4, *E*. *urophylla* ‘GLU4’; G9, *E*. *grandis × E*. *urophylla* ‘GLGU9’; G12, *E*. *grandis × E*. *urophylla* ‘GLGU12’.Treatments: K_0-3_, treatment with 0 mM Al^3+^, pH 3.0; K_4.4–3_, 4.4 mM Al^3+^, pH 3.0; K_0-4_, 0 mM Al^3+^, pH 4.0; K_4.4–4_, 4.4 mM Al^3+^, pH 4.0; CK, control treatment, 0 mM Al^3+^, pH 4.8. Capital letters represent significant differences between treatments within an eucalypt clone at the 0.01 level, small letters are at the 0.05 level, n = 3.

Acid/aluminum treatments reduced the transpiration rates in all eucalypt clones (Fig 3). The change in the transpiration rate was similar to photosynthetic rate, in that G9 > G12 and G3 > G12, while CK > K_0-4_ > K_0-3_ > K_4.4–4_ > K_4.4–3_ for the photosynthetic rate. However, the impact of acid/aluminum on transpiration was smaller than on photosynthesis and the difference in transpiration rates between treatments was less. Except for G4, no significant differences in transpiration rates were observed in the other three clones between the K_0-4_ treatment and the CK. The difference between various treatments in G9 was not significant. Under the K_0-3_ and K_4.4–4_ treatments, significant differences were observed between treatments and the corresponding controls in all four clones. Under the K_4.4–3_ treatment, the transpiration rates in all clones decreased significantly; the transpiration rates in G3, G4, G9 and G12 were 2.25, 1.81, 2.51 and 2.54 μmol × m^-2^ × s^-1^, respectively, which were significantly distinct from the CK and other treatments.

As for the range of change in the respiration rate of different clones, the rate of G9 and G12 decreased little under acidic aluminum stress (0.3~3%), and were 10% lower than the other twoclones. The declines in G3 and G4 under the K_0-3_, K_0-4_ and K_4.4–4_ treatments were almost equal (0.1~3%). In the case of K_4.4–3_, the declines in all clones were the greatest with declines in G3, G4, G9 and G12 of 49.6%, 57.5%, 38.9% and 39.5%, respectively. Meanwhile, in the case of the presence of aluminum when compared with the corresponding CK, the decline in the transpiration rate under conditions of pH 3.0 and 4.0 were higher by 22~25% and 12~19%, respectively, than those without aluminum, showing that low acidity and aluminum ions interferes with normal plant water uptake and transport.

#### Water use efficiency

**Fig 4:**
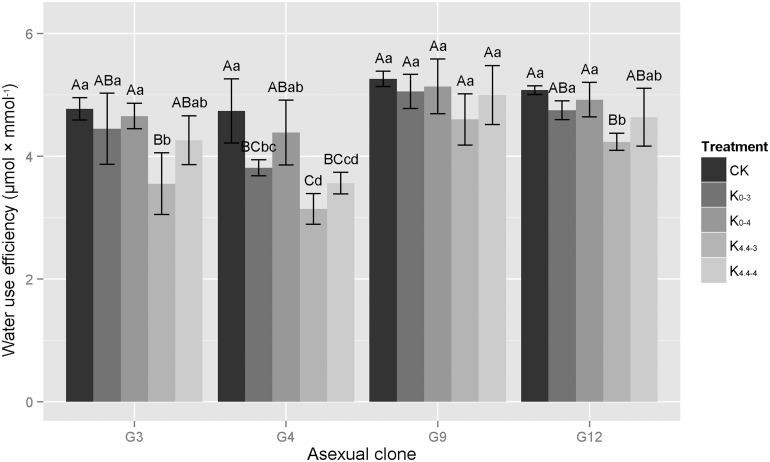
Changes in water use efficiencies of four eucalypt clones under different acid aluminum treatments. G3 represents *E*. *urophylla × E*. *camaldulensis* ‘GLUC3’; G4, *E*. *urophylla* ‘GLU4’; G9, *E*. *grandis × E*. *urophylla* ‘GLGU9’; G12, *E*. *grandis × E*. *urophylla* ‘GLGU12’.Treatments: K_0-3_, treatment with 0 mM Al^3+^, pH 3.0; K_4.4–3_, 4.4 mM Al^3+^, pH 3.0; K_0-4_, 0 mM Al^3+^, pH 4.0; K_4.4–4_, 4.4 mM Al^3+^, pH 4.0; CK, control treatment, 0 mM Al^3+^, pH 4.8. Capital letters represent significant differences between treatments within an eucalypt clone at the 0.01 level, small letters are at the 0.05 level, n = 3.

Plant WUE indicates the fixed amount of CO_2_ a plant produces per unit of water consumption and is an important indicator of plant growth. When the concentration of aluminum increased, the WUE in each of the four clones studied here declined (Fig 4). No significant difference in WUE was observed between the CK and various treatments in G9. In addition, no significant difference in WUE was observed between the CK and the K_0-3_, K_0-4_ and K_4.4–4_ treatments in G3 and G12. Likewise, no significant difference was observed between the K_0-3_, K_0-4_ and K_4.4–4_ treatments, but a significant difference was observed between these treatments and the K_4.4–3_ treatment in G4. As for changes in the magnitude of WUE, without the addition of aluminum ions, at a pH of 3.0 or 4.0, the WUE decline of G4 reached 20% and 7.5%, respectively. However, WUE decreased by only 2.3~6.8% in other three clones at pH 3.0 and pH 4.0. After the addition of aluminum ions, at pH 4.0, the decline in WUE in G9 and G12 were 5.0% and 8.7%, respectively. Similarly, at pH 4.0 the decline in WUE in G3 and G4 was 10.8% and 24.8%, respectively. Additionally, at pH 3.0, all four clones exhibited their lowest WUE when compared to other treatments; the decline in WUE in G3, G4, G9 and G12 was 25.6%, 33.7%, 12.6% and 16.6%, respectively. Therefore, aluminum ions can interfere with the water use of eucalypts.

### Leaf morphological structure

The effects of acid and aluminum on morphological structure of leaves from four *Eucalyptus* clones were also observed. Compared with the CK, the acid and aluminum stress reduced leaves’ thickness on the whole. Table 1 showed variations in morphological structure in detail. The blade thickness of G9 and G12 clones were higher than that of G3 and G4, but there was little difference in the thickness of upper and lower epidermis, palisade and spongy tissues between these four clones under normal growth conditions. After the treatment by acid and aluminum, the thickness of the upper epidermis in G3 and G12 obviously decreased and the thickness of lower epidermis in G4 decreased very significantly. With those same treatments, the thickness of palisade tissue decreased by 28.0% (G3) and 41.4% (G12), while the thickness of lower epidermis in G9 only decreased 8%; the thickness of sponge tissue changed very little. After the acid and aluminum treatments, the palisade tissue/spongy tissue ratio decreased significantly; the difference between treatments became very significant. From the view of tissue tightness and looseness, both tissue tightness and looseness in G9 and tissue looseness in G12 did not change significantly while the change was very significant difference between the other treatments. In particular, the tissue looseness in G4 decreased 36.4%.

**Table 1:** Leaf thickness of four eucalypt clones under acid and aluminum treatments.

Clones^a^	Treatments	Leaf thickness (μm)	Thickness of epidermis (μm)		Thickness of tissue (μm)		Palisade tissue / spongy tissue ratio	Tissue tightness	Tissue porosity
			Upper epidermis	Lower epidermis	Palisade tissue	Spongy tissue			
**G3**	**CK**	220.40 Aa	16.32Aa	12.25 Aa	86.81 Aa	106.31Aa	0.82 Aa	0.39 Aa	0.48 Aa
	**K_4.4–3_**	206.89 Bb	13.45 Bb	11.13 Aa	62.51 Bb	112.86 Bb	0.55Bb	0.30 Bb	0.55 Bb
**G4**	**CK**	197.19 Aa	14.29 Aa	13.04 Aa	86.56 Aa	105.50 Aa	0.82 Aa	0.44 Aa	0.54 Aa
	**K_4.4–3_**	184.16 Bb	12.03 Ab	10.12 Bb	50.70 Bb	116.79 Bb	0.43 Bb	0.28 Bb	0.63 Bb
**G9**	**CK**	198.12 Aa	14.75 Aa	13.81 Aa	88.13 Aa	106.25 Aa	0.83 Aa	0.44 Aa	0.54 Aa
	**K_4.4–3_**	190.75 Bb	13.77 Aa	12.07 Ab	81.06 Bb	108.90 Bb	0.74 Bb	0.42 Aa	0.57 Aa
**G12**	**CK**	197.96 Aa	15.18 Aa	13.92 Aa	86.27 Aa	105.94 Aa	0.81 Aa	0.44 Aa	0.54 Aa
	**K_4.4–3_**	198.43 Aa	13.60 Bb	12.55 Aa	70.92 Bb	109.08 Bb	0.65 Bb	0.36 Bb	0.55 Aa

Capital letters represent significant differences between treatments within an eucalypt clone at the 0.01 level, small letters are at the 0.05 level, n = 3.

^a^ G3, *E*. *urophylla × E*. *camaldulensis* ‘GLUC3’; G4, *E*. *urophylla* ‘GLU4’; G9, *E*. *grandis × E*. *urophylla* ‘GLGU9’; G12, *E*. *grandis × E*. *urophylla* ‘GLGU12’.

## Discussion

### Photosynthetic parameters and aluminum toxicity

In this study low pH and aluminum toxicity resulted in a gradual decrease in the chlorophyll content, the photosynthetic rates, transpiration rates and water use efficiency. Under the same pH, aluminum addition caused more reduction in all photosynthetic parameters, suggesting that aluminum toxicity was certain to inhibit photosynthetic activity of the selected *Eucalyptus* clones. Under aluminum stress, a number of factors are recognized to act on plant photosynthesis, which can be divided into stomatal and non-stomatal factors. Here, we speculate that decrease in photosynthetic parameters might be a synthetic result of multiple factors. For chlorophyll content, it might be indirectly inhibited by aluminum addition, because Al can compete with Mg which is an integral part of chlorophyll molecule to bind the binding sites on plasma membrane of roots, interfering with Mg uptake and transport [35]. Moreover, eucalypts which are fast-growing trees may have an immediate demand for Mg [36].

Except for chlorophyll content, negative effects of aluminum on photosynthetic rate can be explained by changes in photosystem I (PSI) and II (PSII). Al^3+^ was reported to stimulate PSII, catalyze electron flow and O_2_ evolution, but suppress PSI mediated electron transport [37, 38], while under Al stress, electron transport in PSII was inhibited in seedlings of *Citrus reshni* Hort. ex Tanaka [39]. Besides, the major sites of ROS (reactive oxygen species) generation are the reaction centers of PSI and PSII in chloroplast thylakoids in plants [40], as a result, the oxidative stress produced by Al probably causes damage of photosynthetic apparatus.

Observed decrease in transpiration rates might be related to change of stomatal behavior. Previous studies demonstrated that Al stress induced stomatal closure, Smirnov et al. [41] reported that under 0.05 mM Al, changes in stomatal shape coefficient were observed, which directly connected with stomatal closure. The stomatal closure is usually considered as a sign of transpiration inhibition. Early studies indicated that inhibited transpiration was contributed to effect of aluminum on plant growth [42, 43]. In fact, under aluminum toxicity, the plants may have different transpiration performance. Decreased transpiration was found in *Hordeum*, *Scutellaria* and *Populus* [22, 44, 45], which are consistent with our results, but Pereira et al. [21] found the transpiration of citrus rootstocks declined significantly under Al stress.

For water utility efficiency (WUE), the decrease should partly explain photosynthesis reduction. WUE is recognized as an important characteristic reflecting adaptability of plants to environmental stress. Transpiration competes with photosynthesis for water under Al stress, and the water deficiency subsequently restrains photosynthesis. Ali et al. [35] reported that 1 and 10 mM Al treatments decreased WUE of mung bean. Ying and Liu [46] also reported Al decreased both transpiration and WUE of soybean. However, in spite of decreased WUE at 0.1, 0.2, or 0.4 mM Al, Al at 0.05 mM caused increase in WUE of citrus rootstocks [21]. Similarly, Simon et al. [47] observed that under 0.05 mM Al, WUE of one tomato cultivars increased by 56%, whereas the other cultivar was not affected by Al.

Certainly, the previous records also indicated that under Al stress, other non-stomatal limitations play an important role in affecting directly or indirectly plant photosynthesis. For example, harmed chloroplast envelope, carotenoid contents and ratio of chlorophyll a and b were observed under aluminum stress [46, 48]. RuBisCO activity and carboxylation efficiency were inhibited by aluminum [48], negatively effecting on CO_2_ fixation, concentrations of ATPases and GAPases were reduced as well as ATP synthesis in leaves [49]. Otherwise, uptake to nitrogen and phosphorus had negative responses, when plants suffered from aluminum toxicity [50]. Similar to magnesium, irondeficiency caused by aluminum stress constrains chlorophyll synthesis and photochemical efficiency of PSII [51, 52].

### Low pH and aluminum toxicity

Under 4.4 mM aluminum, lower pH (pH 3.0) resulted in greater reduction in photosynthetic parameters than pH 4.0. Aluminum is a major constituent in a wide array of primary and secondary minerals in soil [53], while the soil pH is the one of most important factors controlling the amount of Al^3+^ available for plant uptake in the soil solution. Soil with a pH above 6.0 does not contain toxic levels of aluminum. when the pH begins to drop below pH 6.0, these primary and secondary minerals dissolve to a limited extent, releasing Al into the soil solution, aluminum will become plant available, where it may be hydrolyzed and contribute to soil acidity. When the soil pH is between 5.0 and 5.5, the increase in aluminum in soil solution may be slightly toxic. Below 5.0, soluble Al rises dramatically in nearly all soils there is a very good chance that the soil contains toxic levels of aluminum [54]. As a result, the similar trend in this study was found that the K_4.4–3_ treatment induced the most serious photosynthetic inhibition in all eucalypt clones.

### *Eucalyptus* clones

Plants exhibited different responses because of their different aluminum tolerances. For eucalypts, the photosynthetic rate can be considered as one of the reference indicators for aluminum tolerance of clones. Our findings showed some differences in aluminum stress tolerance existed in different genotypes. For example, photosynthetic and transpiration rates declined more in the genotypes that were susceptible to aluminum stress, G3 and G4, but declined less in resistant genotypes, G9 and G12. This is similar to the effects of aluminum stress on the photosynthetic characteristics of the varieties of soybean [46], maize [55] and barley [22] with different levels of aluminum tolerance.

When plants accumulate aluminum, this will lead to nutritional deficiencies, and these in turn lead to decreased photosynthetic activity [56]. Neusa et al. [57] used acidic aluminum treatments and reported that plants from *Plantago* genus were relatively resistant to aluminum toxicity; their roots easily accumulated aluminum, and aluminum decreased significantly the photosynthetic pigments in *Plantago algarbiensi* with a low soil pH (4.0). However, the photosynthetic system in *Plantago almogravensis* showed better adaptability, indicating that the response of different species to aluminum stress under varied acidic conditions. The preliminary studies conducted by Yang et al. [58] showed that *Eucalyptus* roots accumulated aluminum after treatment of soil with acid aluminum. Meanwhile, the accumulation of aluminum in aboveground parts was relatively low in that study. In addition, the aluminum content in various organs of fast-growing *Eucalyptus* clones and their increase in biomass decreased with enhanced aluminum tolerance, indicating an Al-tolerant genotype might have a strong internal rejection of aluminum or some type of detoxification capability. This occurs because the accumulation of variable amounts of aluminum reduced the photosynthetic rates in various clones. In another report from Yang et al. [32], examining aluminum treatment, survival ratio, growth increment, tissue water content, membrane permeability, proline content and plant morphology of seedlings from the four eucalypt clones were investigated. They showed that clones G9 and G12 had superiority in all indices, except membrane permeability. Combining with this study, G9 and G12 have a higher resistance to aluminum toxicity than G3 and G4.

There is evidence that content of oenothein B isolated from roots of *E*. *camaldulensis* was correlated with Al resistance [59]. Oenothein B formed water-soluble or-insoluble complexes with Al depending on the ratio of oenothein B to Al and could bind at least four Al ions per molecule. Al-induced inhibition of root elongation was completely alleviated by treatment with exogenous oenothein B, which indicated the capability of oenothein B to detoxify Al. Oenothein B was localized mostly in the root symplast of *E*. *camaldulensis*. Different *Eucalyptus* species may release distinctive kinds and amounts of organic acid anions following Al^3+^ exposure, the three organic acid anions form complexes with Al^3+^ with the following order of strength: citrate > oxalate > malate, resulting in differences in Al-resistance between *Eucalyptus* species [6, 8, 60]. Unfortunately, due to lacking of more physiological and biochemical evidences, current documents fail to directly and in-depth elucidate Al-resistant mechanism of G9 and G12, but it seems to direct the further work for selecting and breeding excellent *Eucalyptus* clones with Al-resistance in the areas which suffer from Al toxicity.

### Leaf morphology

At the macro-morphological level, Yang et al. [32] found Al-induced distortion of leaves and buds of *Eucalyptus* seedlings. In this study aluminum treatments caused *Eucalyptus* seedlings to form thinner tissues than seedlings without aluminum treatments; the decreased number of mesophyll cells became one of the causes leading to a significant reduction in the photosynthetic and transpiration rates. Increased tissue tightness and reduced porosity may enhance leaf resistance to aluminum stress by reducing the water absorption capacity in roots of seedlings; cell dehydration resulted in reduced leaf thickness [61]. Because palisade tissue contains more chloroplasts than spongy mesophyll tissue, damage to palisade tissue also will inevitably have an effect on the efficiency of leaf photosynthesis. Under high Al concentration, damage of mesophyll cells of oilseed rape was observed [62]. de Almeida et al. [63] found that after Al stress exposure, leaf issue thickness of cacao seedlings increased. In a report from Gomes et al. [64], leaf epidermis thickness on the adaxial and abaxial of *Brachiaria decumbens* (signal grass) increased, but in study of McQuattie and Schier [65], size of leaf mesophyll cells of pitch pine seedlings reduced. Results from Sridhar et al. [66] and Zhao et al. [67] showed that collapse of palisade and spongy parenchyma cells with intense vacuolization induced by toxic metals.

In our present study we speculate that on the one hand, high concentration of aluminum might restrain uptake, transport and accumulation of nutrition elements (N, P, Ca, Mg and Fe) [17, 68–70], which commonly play important roles in biosynthesis and functioning. On the other hand, a reduction in photosynthetic activity might be responsible for less accumulation of photosynthetic products in leaves. The selected leaves of *Eucalyptus* were the positions which grow fastest and were very young, and cell development and growth are most active, shortages of inorganic and organic nutrients possibly exerted destructive effects on leave micro-morphology. Furthermore, what should be noted that ROS induced by Al toxicity might be harmful to structure of membranes and organelles, causing leaf cell injury [71].

Overall, acid aluminum stress altered photosynthetic physiology as well as biomass accumulation of *Eucalyptus* seedlings [32], probably also exerting potential impacts on productivity of eucalypt plantations. Therefore, comparing several *Eucalyptus* clones may bring an insight into growth performance of different genotypes, and provide physiological information for selecting clones and developing new *Eucalyptus* breeds.

## Supporting Information

S1 File Supporting Tables. Table A. Data of chlorophyll content. Table B. Data of net photosynthetic rate. Table C. Data of transpiration rate. Table D. Data of leaf morphological structure.
(XLSX)
